# ﻿*Calothecanigromaculata* species-group from sub-Saharan Africa with descriptions of two new species from KwaZulu-Natal (Chrysomelidae, Galerucinae, Alticini)

**DOI:** 10.3897/zookeys.1084.73175

**Published:** 2022-01-28

**Authors:** Paola D’Alessandro, Mattia Iannella, Elizabeth Grobbelaar, Maurizio Biondi

**Affiliations:** 1 Department of Health, Life and Environmental Sciences, University of L’Aquila, Via Vetoio, I-67100 L’Aquila, Italy University of L'Aquila L'Aquila Italy; 2 Biosystematics Division, ARC-Plant Protection Research Institute, Private Bag X134, Queenswood, Pretoria 0121, South Africa ARC-Plant Protection Research Institute Pretoria South Africa

**Keywords:** Afrotropical region, *Calothecacarolineae* sp. nov., *C.nigromaculata*, *C.wanati* sp. nov., flea beetles, leaf beetles, Republic of South Africa

## Abstract

*Calotheca* Heyden is a flea beetle genus with a largely sub-Saharan distribution and currently comprising 34 species. The examination of new material is revealing an increase in species richness and intraspecific variability. *Calothecacarolineae***sp. nov.** and *C.wanati***sp. nov.**, both from KwaZulu-Natal in the Republic of South Africa, are here described and attributed to the *C.nigromaculata* (Jacoby) species group, mainly based on genitalic characters. Photographs of the main diagnostic characters are provided, including the habitus, median lobe of the aedeagus, and spermatheca. Information on the geographic distribution and host plants of these species is also provided.

## ﻿Introduction

*Calotheca* Heyden, 1887 is a flea beetle genus (Chrysomelidae, Galerucinae, Alticini) that is widespread in sub-Saharan Africa where is particularly common in the eastern and southern parts, with some records from Israel and the Arabian Peninsula (Biondi and D’Alessandro 2010a, 2012; Biondi et al. 2017; D’Alessandro et al. 2018a). Up to now this genus comprised 32 species, mainly associated with plants in the family Anacardiaceae, particularly *Searsia* spp., which are distributed in several different forest and savannah environments (D’Alessandro et al. 2018a, 2020, 2021; Iannella et al. 2021). The new material under examination is revealing an increase in species richness and intraspecific variability. Since the knowledge of the Afrotropical flea beetle fauna is far from exhaustive, unexpected diversity is often discovered within genera (e.g. Biondi and D’Alessandro 2008, 2010b), new genera are recognized (e.g. Biondi and D’Alessandro 2013a), or genera are recorded for the first time from the Afrotropical region (e.g. Biondi and D’Alessandro 2013b). *Calotheca* was identified as a monophyletic group based on a phylogenetic analysis which included *Blepharidarhois* (Forster, 1771) from North America among the outgroups (Biondi et al. 2017). Even though the Afrotropical species of *Calotheca* are the most closely related to the North American *B.rhois*, they are significantly separated from it, and supported by two synapomorphies: frontal grooves sinuate and deeply impressed, extending approximately from the dorsal ocular margin to the interantennal space, and femora strongly punctured. The main diagnostic characters for *Calotheca*, compared to the closely related African genus *Blepharidina* Bechyné, 1968, are the sinuate and deeply impressed frontal grooves, extending approximately from the dorsal ocular margin to the inter-antennal space; and the punctate lateral striae on the pronotum, which run from the anterior margin onto the disc, and are straight, curved, or L- or C-shaped. Some species also have short lateral longitudinal furrows and/or small dimples close to the pronotal base (Biondi et al. 2017, 2019; D’Alessandro et al. 2018b, 2019). In this paper we review, based on newly examined material, the distribution and ecological data of *Calothecanigromaculata* (Jacoby, 1888) from southern Africa. We also describe two new species, *Calothecacarolineae* sp. nov. and *C.wanati* sp. nov., both from KwaZulu-Natal in the Republic of South Africa. The three species are attributed to the *Calothecanigromaculata* species group, based on the morphology of the pronotum and spermatheca.

## ﻿Materials and methods

Material examined consists of 212 dried pinned specimens, preserved in the institutions listed below in the “Abbreviations” section. The specimens were examined, measured, and dissected using a Leica M205C stereomicroscope. Photographs were taken using a Leica DFC500 camera and compiled using Zerene Stacker software, v. 1.04. Scanning electron micrographs were taken using a Hitachi TM-1000. Terminology follows D’Alessandro et al. (2016) for the median lobe of aedeagus and spermatheca. Geographic coordinates for the localities were reported in degrees and minutes format using the WGS84 datum; information included in square brackets were added to the label data by the authors and using the Google Earth website for coordinates and geographic information. Abbreviations for the depositories follow the list on the following website: The Insect and Spider Collections of the World (Evenhuis 2021). Chorotypes follow Biondi and D’Alessandro (2006).

### ﻿Abbreviations

**Collections and depositories. BAQ**: Italy, University of L’Aquila, Collection of M. Biondi; **MNHN**: France, Paris, Muséum National d’Histoire Naturelle; **NHMUK**: United Kingdom, London, The Natural History Museum; **NMPC**: Czech Republic, Prague, National Museum (Natural History); **SANC**: South Africa, Pretoria, South African National Collection of Insects; **UWCP**: Poland, Wrocław, University of Wroclaw; and **ZSM**: Germany, München [= Munich], Zoologische Staatssammlung.

**Biometrics. LA**: numerical sequence from base to apex proportional to the length of each antennomere; **LAED**: length of aedeagus; **LAN**: length of antennae; **LB**: total body length (from apical margin of head to apex of elytra); **LE**: length of elytra; **LP**: medial length of pronotum; **LSP**: maximum length of spermatheca, including ductus; **WE**: maximum width of elytra combined; **WP**: maximum width of pronotum.

**Distribution. KZN**: KwaZulu-Natal; **LIM**: Limpopo; **MPU**: Mpumalanga; **WCape**: Western Cape; [?]: unknown locality.

## ﻿Results

### ﻿ Calotheca carolineae sp. nov.

Taxon classificationAnimaliaPoalesPoaceae

1A483E57-DAE1-5312-9874-649C1AE19945

http://zoobank.org/73C82870-1A5C-43E0-A230-CC0B06C01A0D

**Figure 1.**
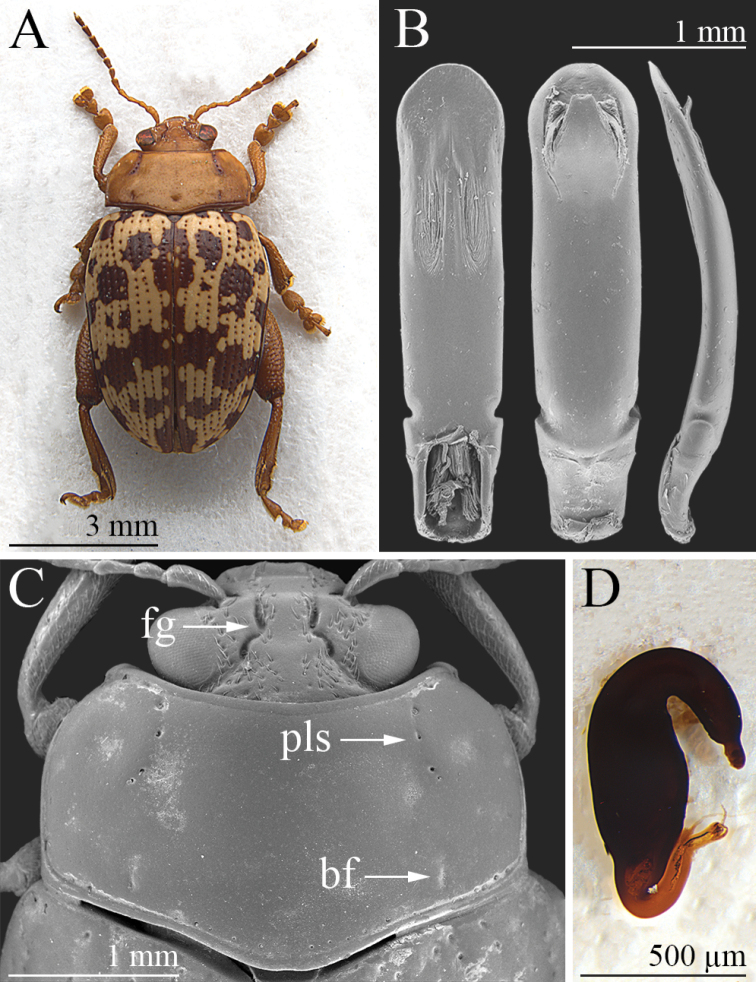
*Calothecacarolineae* sp. nov. **A** habitus, ♂ (holotype, KZN, Kosi Bay Nature Reserve) **B** median lobe of aedeagus, from left to right in ventral, dorsal, and lateral view (KZN, Sodwana Bay Park) **C** head, and pronotum, ♂ (KZN, Sodwana Bay Park) **D** spermatheca (KZN, Sodwana Bay Park). Abbreviations: bf = basal furrow; fg = frontal groove; pls = punctate lateral stria.

Figures 1A–D , 4 , 5

#### Type material.

***Holotype*** ♂: Republic Of South Africa: KZN: Kosi Bay Mouth Nature Reserve, 26°53'31"S, 32°52'39"E, c. 0 m, 24.i.2006, adults collected on *Allophylusnatalensis* (Sapindaceae), C. Chaboo & E. Grobbelaar leg. (SANC). ***Paratypes***: Republic Of South Africa: 1♂1♀, same data as for holotype (SANC); 1♂1♀, KZN: Kosi Bay Nature Reserve, 26°58'S, 32°48'E, 50 m, 08-11.ii.1990, E. Grobbelaar leg., beaten off *Ozoroaobovata* (Oliv.) R.&A. Fern. (Anacardiaceae) (SANC); 2♂5♀, Natal [KZN]: Sodwana Bay Park, 27°32'S, 32°41'E [27°32'25"S, 32°40'37"E], 9–11.xi.1986, D. D’Hotman & A. Nel leg. (SANC).

#### Diagnosis.

**Figure 2.**
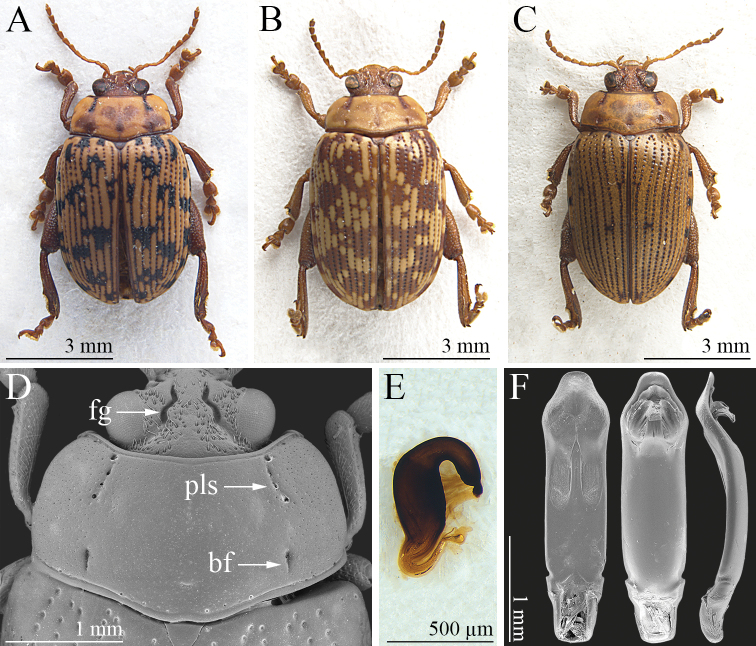
*Calothecanigromaculata*. **A** habitus, ♂ (KZN, Tembe Elephant Park, Research Camp) **B** ibid, ♂ (MPU, Mapoch’s Caves) **C** ibid, ♂ (KZN, Vryheid Hill Nature Reserve) **D** head, and pronotum, ♂ (MPU, Mariepskop base Picnic Site at Blyde River) **E** spermatheca (KZN, Estcourt) **F** median lobe of aedeagus, from left to right in ventral, dorsal, and lateral view (MPU, Pretoriuskop). Abbreviations: bf = basal furrow; bfg = frontal groove; pls = punctate lateral stria.

**Figure 3.**
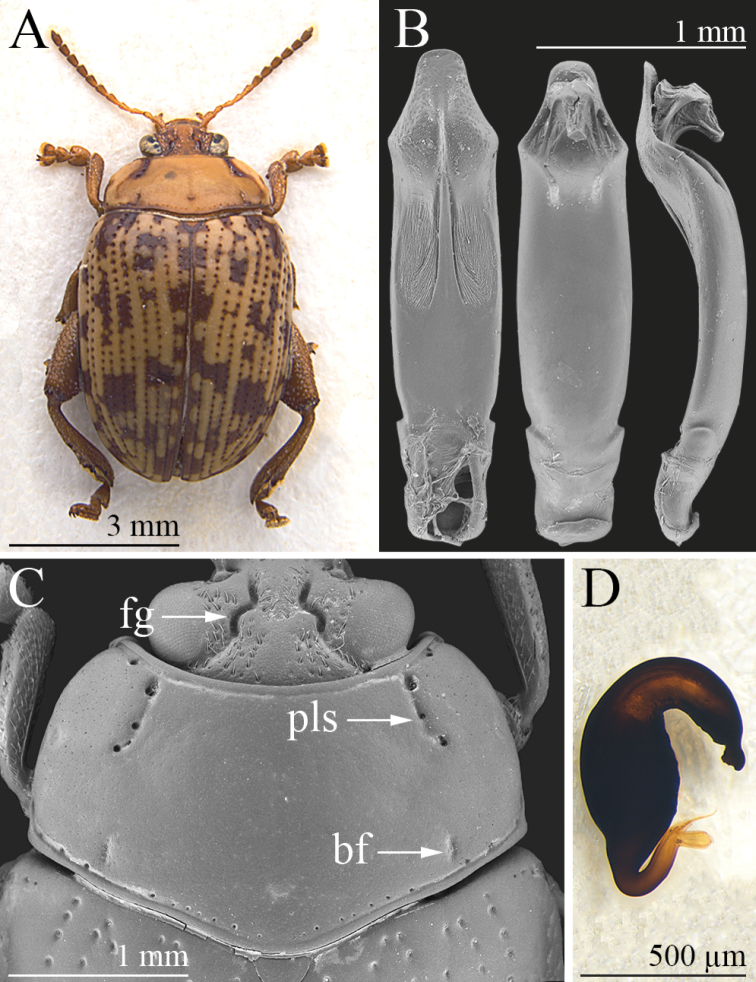
*Calothecawanati* sp. nov. **A** habitus, ♂ (holotype, KZN, Ubombo Mountain Nature Reserve) **B** median lobe of aedeagus, from left to right in ventral, dorsal, and lateral view (KZN, Ubombo Mountain Nature Reserve) **C** head, and pronotum, ♂ (KZN, Ubombo Mountain Nature Reserve) **D** spermatheca (KZN, Ubombo Mountain Nature Reserve). Abbreviations: bf = basal furrow; fg = frontal groove; pls = punctate lateral stria.

**Figure 4.**
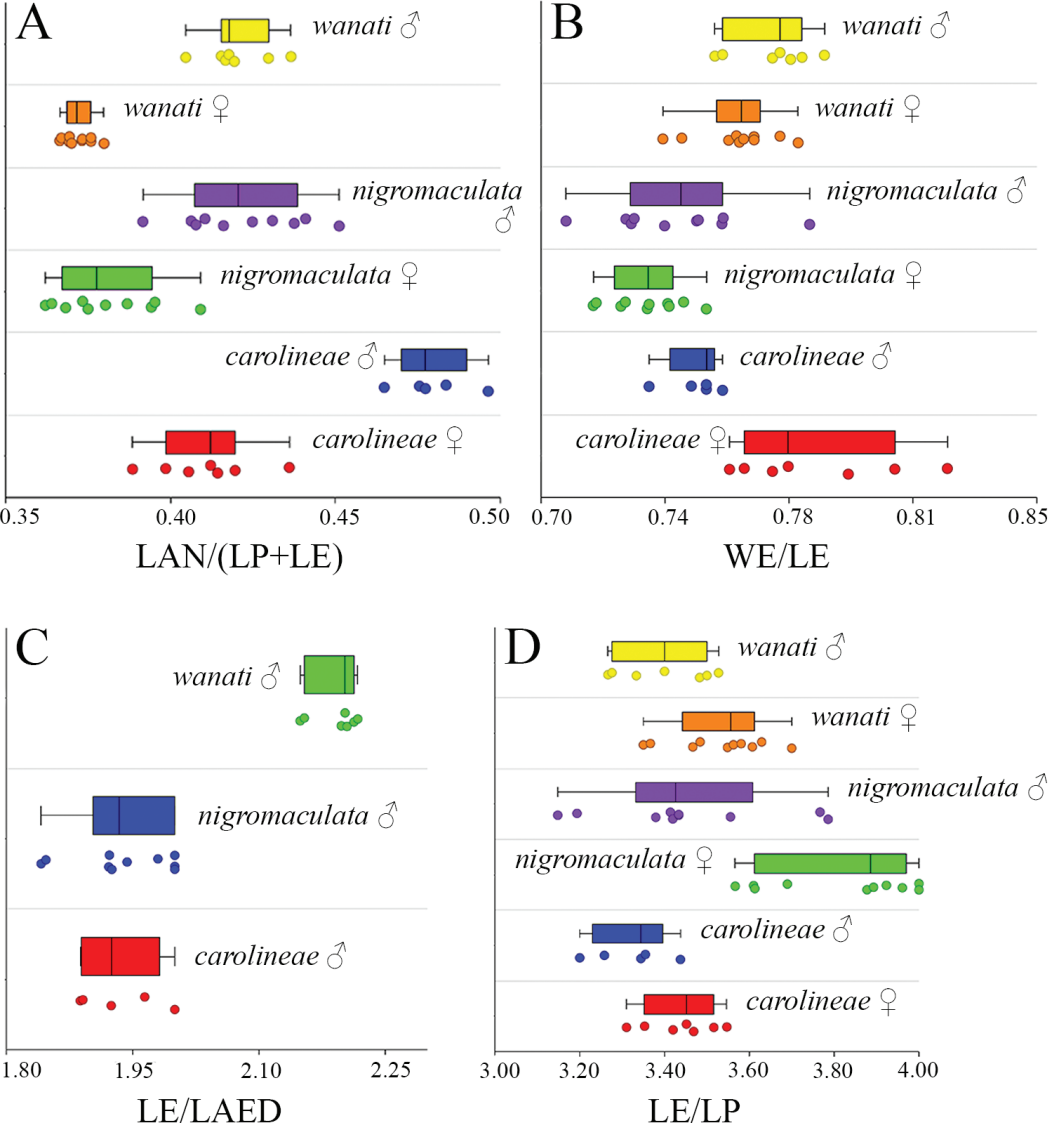
Histograms of morphometric variables in the *Calothecanigromaculata* species group. Abbreviations: LAED = length of aedeagus; LAN = length of antennae; LE = length of elytra; LP = medial length of pronotum; WE = maximum width of elytra combined; WP =maximum width of pronotum.

*Calothecacarolineae* sp. nov. displays major similarities with *C.nigromaculata* and *C.wanati* sp. nov., more so than with other known *Calotheca* species. It is mainly characterized by the differently shaped median lobe of the aedeagus (Figs 1B, 2F, 3B) which is not sinuate in lateral view, and widely rounded in the apical part in ventral view (in *C.nigromaculata*, even though quite variable, it is curved in proximal 1/2 and distinctly sinuate in apical 1/2 in lateral view, and produced and subtruncate apically in ventral view; in *C.wanati* sp. nov. it is curved in proximal 1/2 and more distinctly curved in apical 1/2 in lateral view, and produced and sub-rhomboidal in the apical 1/4, with prominent angulate lateral projections in ventral view). Other differences include: color of the dorsal integument with more clearly defined and generally larger colored elytral patches, reddish-brown on a yellow background (smaller and more confused, from brown to black, in *C.nigromaculata* and *C.wanati* sp. nov.) (Figs 1A, 2A–C, 3A); punctate lateral pronotal striae less dark (Figs 1A, 2A–C, 3A); antennae generally longer with LAN/(LE+LP) = 0.48 ± 0.01 in male, and = 0.41 ± 0.01 in female (in *C.nigromaculata*LAN/(LE+LP) = 0.42 ± 0.02 in male, and = 0.38 ± 0.02 in female; in *C.wanati* sp. nov. LAN/(LE+LP) = 0.42 ± 0.02 in male, and = 0.37 ± 0.02 in female) (Fig. 4A); shape of the pronotum, straight laterally in basal 2/3 and abruptly incurved in apical 1/3 (slightly curved in basal 2/3 and distinctly incurved in apical 1/3 in *C.nigromaculata* and *C.wanati* sp. nov.) (Figs 1C, 2D, 3C).

#### Description of the holotype

**(♂).** Body elliptical in dorsal view (Fig. 1A), rather convex in lateral view; total body length (LB) = 7.00 mm; maximum pronotal width in the apical third (WP = 3.05 mm); maximum width of elytra in the basal 1/3 (WE = 3.85 mm). Head and antennae pale brown, with antennomeres 6–11 slightly darker (Fig. 1A); head surface microreticulate, with evident setiferous punctures on most of the vertex and part of the frons (Fig. 1C); frontal grooves sinuate, deeply impressed, extending approximately from the dorsal ocular margin to the inter-antennal space (Fig. 1C); eyes elliptical, clearly elongate; antennae slightly shorter than half the body length (LAN = 3.15 mm; LAN/LB = 0.45); LA: 100:44:61:63:73:68:73:68:73:63:88. Pronotum (Fig. 1A, C) slightly convex, pale brown; punctate lateral stria, basal dimples and margins slightly darker; distinctly transverse in dorsal view (LP = 1.53 mm; WP/LP = 2.00), sub-rectangular, distinctly incurved laterally in the anterior third only; surface microreticulate, with minute punctation; anterolateral surface with very shallow depressions; lateral punctate striae distinctly impressed, C-shaped and slightly curved on the disc; some additional sparse punctures along the pronotal margins; basal furrows short and moderately incised; anterior and basal margins finely bordered, lateral margins thickened slightly, poorly visible in dorsal view; anterior angles distinctly prominent, moderately swollen; posterior angles obtuse. Scutellum brown, sub-triangular. Elytra (Fig. 1A) pale yellow, with irregular wide brown patches and dark punctation; elytra moderately elongate (LE = 5.10 mm; WE/LE = 0.75; LE/LP = 3.34), distinctly sinuate laterally, jointly rounded apically; lateral margin narrow, visible in dorsal view; elytral punctation arranged in single regular rows formed by distinctly impressed punctures; interstriae flat on the elytral disc, with finely microreticulate and sparsely micropunctate surface; humeral calli indistinctly raised. Macropterous. Basal pro- and mesotarsomeres clearly enlarged (Fig. 1A). Underside pale brown; apical abdominal ventrite without preapical sculptures or impressions. Median lobe of the aedeagus (LAED = 2.65 mm; LE/LAED = 1.92) (Fig. 1B) with subparallel sides in ventral view, widely rounded in the apical part; ventral surface with a pair of lateral U-shaped depressions with a wrinkled surface in the distal half, and evident punctation apically; moderately and evenly curved up to the apex in lateral view; dorsal ligula short, formed by a wide subtriangular, apically truncate median lobe, and two narrow lateral lobes.

#### Variability.

Males (*n* = 5; mean ± standard deviation, range): LE = 5.21 ± 0.17 mm (5.05 ≤ LE ≤ 5.50 mm); WE = 3.89 ± 0.14 mm (3.70 ≤ WE ≤ 4.10 mm); LP = 1.57 ± 0.04 mm (1.55 ≤ LP ≤ 1.63 mm); WP = 3.14 ± 0.09 mm (3.05 ≤ WP ≤ 3.25 mm); LAN = 3.25 ± 0.9 mm (3.15 ≤ LAN ≤ 3.35 mm); LAED = 2.70 ± 0.08 mm (2.60 ≤ LAED ≤ 2.80 mm); LB = 6.83 ± 0.31 mm (6.45 ≤ LB ≤ 7.15 mm); LE/LP = 3.32 ± 0.09 (3.20 ≤ LE/LP ≤ 3.44); WE/WP = 1.24 ± 0.02 (1.21 ≤ WE/WP ≤ 1.26); WP/LP = 2.00 ± 0.03 (1.97 ≤ WP/LP ≤ 2.03); WE/LE = 0.75 ± 0.01 (0.73 ≤ WE/LE ≤ 0.75); LAN/LB = 0.48 ± 0.02 (0.45 ≤ LAN/LB ≤ 0.51); LE/LAED = 1.93 ± 0.05 (1.89 ≤ LE/LAED ≤ 2.00). Females (n = 7; mean ± standard deviation; range): LE = 5.40 ± 0.31 mm (4.80 ≤ LE ≤ 5.70 mm); WE = 4.23 ± 0.24 mm (3.95 ≤ WE ≤ 4.60 mm); LP = 1.57 ± 0.08 mm (1.45 ≤ LP ≤ 1.70 mm); WP = 3.18 ± 0.15 mm (2.90 ≤ WP ≤ 3.38 mm); LAN = 2.87 ± 0.14 mm (2.70 ≤ LAN ≤ 3.05 mm); LSP = 0.74 ± 0.05 mm (0.68 ≤ LSP ≤ 0.80 mm); LB = 6.79 ± 0.51 mm (5.85 ≤ LB ≤ 7.25 mm); LE/LP = 3.43 ± 0.09 (3.31 ≤ LE/LP ≤ 3.55); WE/WP = 1.33 ± 0.04 (1.29 ≤ WE/WP ≤ 1.36); WP/LP = 2.03 ± 0.03 (1.99 ≤ WP/LP ≤ 2.06); WE/LE = 0.78 ± 0.02 (0.76 ≤ WE/LE ≤ 0.82); LAN/LB = 0.42 ± 0.03 (0.39 ≤ LAN/LB ≤ 0.47); LE/LSP = 7.28 ± 0.29 (6.94 ≤ LE/LSP ≤ 7.85). Paratypes very similar in shape, sculpture, and color to the holotype. Basal furrows and punctate lateral striae weakly to distinctly impressed. Female with basal pro- and mesotarsomeres less enlarged than in male. Spermatheca (Fig. 1D) sub-fusiform and elongate basally, narrowing towards the ductus attachment; distal part distinctly curved and about 2/3 the basal part in length, with a distinct appendix; ductus basally inserted, thickset, short, uncoiled, roughly U-shaped.

#### Etymology.

The specific epithet is a noun in the genitive case after our friend Caroline S. Chaboo (University of Nebraska-Lincoln, Nebraska, USA), one of its collectors and appreciated expert of chrysomelid Coleoptera.

#### Distribution.

**Figure 5.**
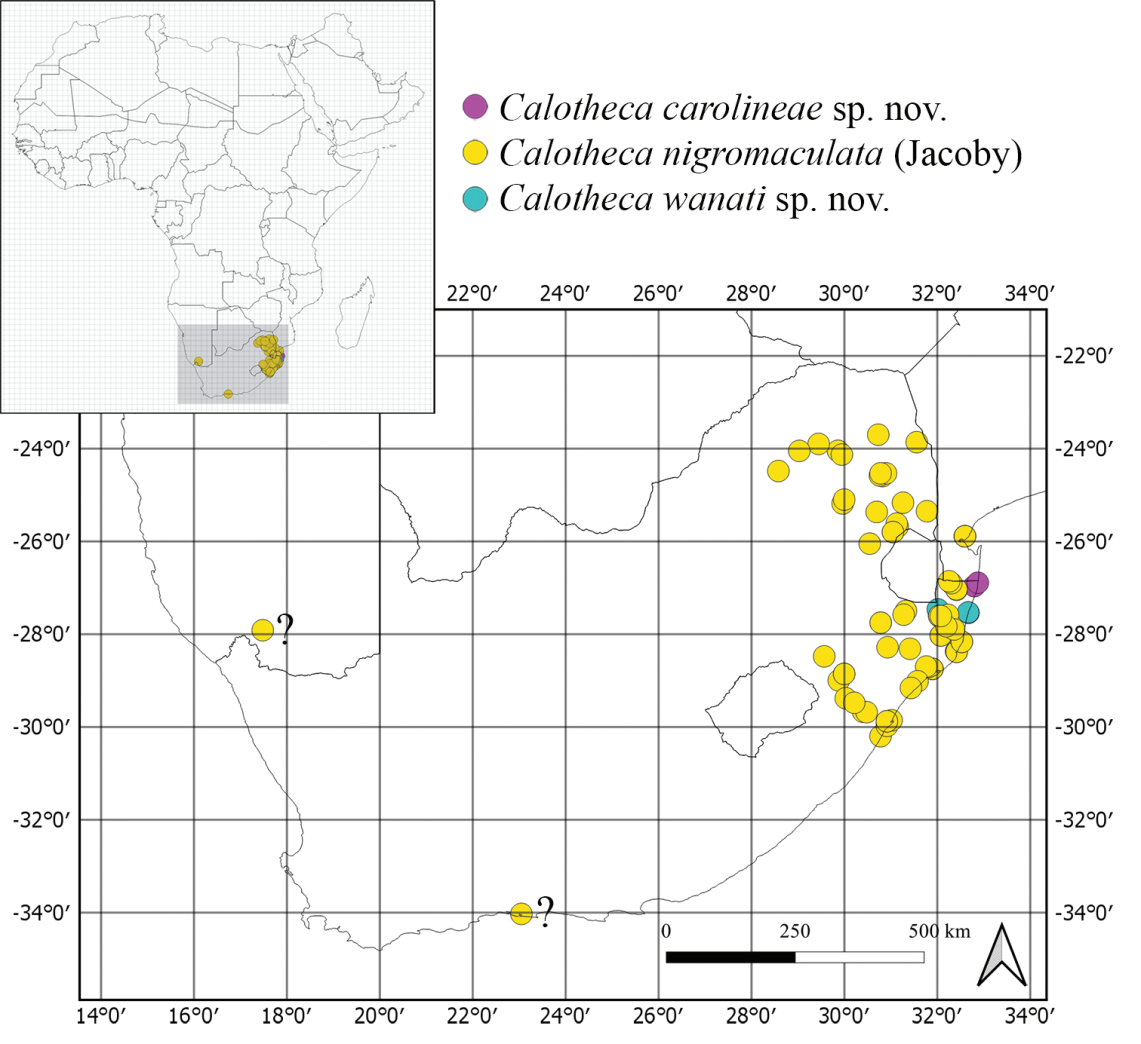
Distribution of the *Calothecanigromaculata* species group.

Republic of South Africa (KZN) (Fig. 5). Chorotype: Southern-Eastern African (SEA).

#### Ecological notes.

Adults were collected in November, January, and February, between 0–50 m a.s.l., on *Allophylusnatalensis* (Sapindaceae) and *Ozoroaobovata* (Anacardiaceae).

### ﻿ Calotheca nigromaculata

Taxon classificationAnimaliaPoalesPoaceae

(Jacoby)

7FD47C3D-D55A-555D-808B-70BA507A9B0E

Figures 2A–F , 4 , 5

Blepharida nigromaculata Jacoby, 1888: 194
Calotheca nigromaculata (Jacoby) Biondi et al. 2017: 121 (pars)

#### Type material examined.

***Lectotype*** ♂: [Mozambique]: Delagoa B[ay] [Maputo Bay, 25°53'31"S, 32°36'18"E], [R.] Monteiro [leg.], Jacoby Coll., 1909-28a (NHMUK) (M. Biondi des. 2017). ***Paralectotype***: 1♂; same data as for lectotype (NHMUK).

#### Additional material examined.

Mozambique: 10 specimens, Delagoa Bay [Maputo Bay, 25°53'31"S, 32°36'18"E], [R.] Monteiro [leg.], ex. coll. R. Oberthur (MNHN); 2 specimens, ibid, 1885 (MNHN). Republic Of South Africa: [KZN]: 3 specimens, Hluhluwe Game Reserve, 28°02'S, 32°05'E, 4–6.ii.1994, U. Göllner leg. (ZSM); 1 specimen, ibid, 4–7.ii.1994 (ZSM); 3 specimens, Natal [KZN]: Itala Game Reserve, Thalu River, 27°30'S, 31°20'E, 27.i.1994, U. Göllner leg. (ZSM); 1 specimen, Natal [KZN]: Itala Game Reserve, Louwsburg, 27°35'S, 31°17'E, 10–23.xii.1992, F. Koch leg. (BAQ); 1 specimen, Natal [KZN]: Santa Lucia [28°22'21"S, 32°24'51"E], 29.x.1981, Klapperich leg. (BAQ); 1 specimen, [KZN]: St. Lucia Estuary, 22.x.[19]66, G. du Plessis leg., (SANC); 1 specimen, ibid, 24.x.[19]66 (SANC); 1 specimen, KwaZulu-Natal: Mkuze Natural Reserve, 27°37'S, 32°03'E, 100 m, 16.xi.1988, Colonnelli leg. (BAQ); 1 specimen, Zululand [KZN]: Mkuzi [Mkuze, 27°36'24"S, 32°02'53"E], xii.1945, DDT Killed, DDT No. 153; 7/15; Imp. Inst. Ent. Coll.No. 10519 (SANC); 7 specimens, KZN: Mkuzi Game Res.[erve], c. 2 km NE Mantuma Rest Camp, 27°35'06"S, 32°14'14"E, c. 69 m, 21.i.2006, adults beaten off cf. *Rhusgueinzii* (Anacardiaceae), C. Chaboo & E. Grobbelaar leg. (SANC); 2 specimens, [KZN]: King[s]burgh, 18km S, 30°05'S, 30°47'E, 24.ii.1989, B.[=E.] Grobbelaar & E. v.d. Linde leg. (SANC); 1 specimen, Natal [KZN]: Cape Vidal, 28°10'S, 32°32'E, 15.xi.1986, D. D’Hotman & A. Nel leg. (SANC); 2 specimens, ibid, 13.i.1981, I.M. Millar leg. (SANC); 3 specimens, KZN: Tembe Elephant Park, Research Camp, 27°02'40"S, 32°25'17"E, c. 100 m, 25-26.i.2006, adults beaten off cf. *Allophylusdecipiens* (Sapindaceae), C. Chaboo & E. Grobbelaar leg. (SANC); 3 specimens, KZN: Tembe Elephant Park, Sihangwane Area, 27°02'S, 32°25'E, 100 m, 01.ii–04.ii.1996, collected from *Rhus* sp. (Anacardiaceae), E. Grobbelaar leg. (SANC); 8 specimens, Natal [KZN]: Estcourt, 29°00'S, 29°53'E, 25.ii.1984, R. Oberprieler & C.G.E. Moolman leg. (SANC); 1 specimen, Natal [KZN]: Pietermaritzburg, Ukulinga Station [29°40'27"S, 30°24'31"E], 3.x.1983, A. Freidberg leg. (DG Furth coll) (BAQ); 1 specimen, Natal [KZN]: S Coast, Umkomaas [30°12'06"S, 30°46'57"E], 11.x.1983, A. Freidberg leg. (DG Furth coll) (BAQ); 1 specimen, [KZN]: Isipingo, Nat., [29°58'58"S, 30°55'20"E], ii.1896 (NHMUK); 2 specimens, KZN: Ndumo Game Reserve, c. 1 km NE Rest Camp, 26°54'07"S, 32°18'20"E, c. 80 m, 28.i.2006, collected by beating, C. Chaboo & E. Grobbelaar leg. (SANC); 4 specimens, KZN: Ndumo Game Reserve, Fig. Tree Forest, 26°51'39"S, 32°15'32"E, c. 42 m, 29.i.2006, adults beaten off *Rhusgueinzii* (Anacardiaceae), C. Chaboo & E. Grobbelaar leg. (SANC); 6 specimens, KZN: Vryheid Hill Nature Res.[erve], Ntinginono Eco Centre, 27°45'14"S, 30°47'11"E, c. 1259 m, 30.i–02.ii.2007, E. Grobbelaar leg. (SANC); 6 specimens, ibid, adults beaten off *Rhus* sp. (Anacardiaceae) (SANC); 1 specimen, KZN: Empangeni, 28°45'S, 31°54'E, 152 m, xii.1999, P.E. Reavell leg. (SANC); 1 specimen, KZN: Lewomba, SE 28 31 Da [Lewomba Miss., Empangeni, 28°44'54"S, 31°53'53"E], 20.iv.1979, R. Oberprieler leg. (SANC); 1 specimen, KZN: Lugwavana [?], SE 27 31 Bb, 1.i.1980, on forest vegetation, R. Oberprieler leg. (SANC); 1 specimen, KZN: Ingwavuma, Mac’s Pass, SE 27 31 Bb [28°44'54"S, 31°53'53"E], 13.i.1980, on vegetation, R. Oberprieler leg. (SANC); 1 specimen, Natal [KZN]: Lynnfield Park, 13km SE Pietermaritzburg, 29°41'S, 30°29'E, 28–30.iii.1989, A.E. Whittington leg. (SANC); 1 specimen, NTL [KZN]: Kuleni Farm, Hluhluwe, 27°54'S, 32°22'E, 13–14.ii.1990, N. Verheijen leg. (SANC); 1 specimen, Natal [KZN]: Balgowan, 29°23'S, 30°02'E, 26.ii.1984, R. Oberprieler & C.G.E. Moolman leg. (SANC); 1 specimen, KZN: Ntinini Nature Reserve, 28°17'S, 30°56'E, 1015 m, 16.xi.2010, collected by sweeping through very short grass with various forbs, some flowering, R. Stals leg. (SANC); 2 specimens, KZN: Nyala Game Ranch [28°42'S, 31°46'E], 16.xii.1980, R. Oberprieler leg. (SANC); 1 specimen, KZN: Intendele Game Ranch, nr Bayala, 27°50'S, 32°12'E, 07.i.2000, ex *Rhus* sp.1 (Anacardiaceae), C.N. Duckett leg. (SANC); 4 specimens, Natal [KZN]: Dr. Martin (NMPC); 6 specimens, ibid (MNHN); 1 specimen, ibid (NHMUK); 1 specimen, ibid, Zululand (NMPC); 1 specimen, ibid (SANC); 1 specimen, [KZN]: Howick [29°29'21"S, 30°12'60"E], 1901, J.P. Cregoe leg. (NHMUK); 2 specimens, [KZN]: Durban [29°51'31"S, 31°01'18"E], x.1896, J.P. Cregoe leg. (NHMUK); 2 specimens, ibid, viii.[19]20, A.F.J. Gedye leg. (NHMUK); 1 specimen, [KZN]: Durban, The Bluff [29°56'08"S, 31°00'07"E], 15.x.1931, Mrs L. Ogilvie leg. (NHMUK); 1 specimen, Natal [KZN]: Weenen [28°51'31"S, 30°00'12"E], xii.1926, H.P. Thomasset leg. (NHMUK); 4 specimens, ibid, i.1927 (NHMUK); 1 specimen, ibid, xi.1927 (NHMUK); 2 specimens, ibid, iii-iv.1925 (NHMUK); 1 specimen, Zululand [KZN]: Gingindhlovu [29°01'S, 31°35'E], 9.vi.1926, R.E. Turner leg. (NHMUK); 2 specimens, Natal [KZN]: Lower Tugela [29°09'50"S, 31°26'16"E], E. Reynolds leg. (NHMUK); 1 specimen, Natal [KZN]: Malvern [Malvern, Queensburgh, 29°53'S, 30°55'18"E] iii.1897, G.A.K. Marshall leg. (NHMUK); 1 specimen, ibid, xii.1899, J.P. Cregoe leg. (NHMUK); 4 specimens, KwaZulu-Natal: between Colenso and Weenen Game Reserve, 28.48S, 29.57E [28°28'48"S, 29°34'12"E], 900 m, 4.iii.1998, P. Audisio, M. Biondi & M. Zapparoli leg. (BAQ); 19 specimens Natal [KZN]: Ulundi [28°19'S, 31°25'E], 22.i.1994, A. Poll leg. (ZSM); 1 specimen (NE), KwaZulu-Natal: Ubombo Mountain Nat. Res., –27.6100S/32.0802E [27°36'36"S, 32°04'49"E], 110 m, beating, 30.xi.2012, M. Wanat leg. (UWCP); 2 specimens, Tvl. [LIM]: Hans Merensky Nat.[ure] Res.[erve], 23°42'S, 30°44'E, 23–25.i.1987, collected by beating, B.[=E.] Grobbelaar leg. (SANC); 1 specimen, Limpopo: Strydpoortberge Pass, hill slope, S24 02.741 E29 52.198, [24°02'44"S, 29°52'12"E], 1650 m, 21.ii.2007, P. Audisio & M. Biondi leg. (BAQ); 1 specimen, [LIM]: Pietersburg [Polokwane], 24°14'40"S, 29°15'30"E [23°53'55"S, 29°27'01"E], 18.ii.1989, F.J. Joubert leg. (SANC); 5 specimens, [LIM]: Mathlari, Nas. K.W. [Kruger National Park], 17.iii.1970, H.A.D. van Schalkwyk leg. (SANC); 4 specimens, NProv [LIM]: Thabaphaswa (Groenkom Farm), near Potgietersrus, 24°03'S, 29°02'E, 21–23.ii.2001, adults and larvae collected from *Rhusleptodictya* (Anacardiaceae), E. Grobbelaar leg. (SANC); 1 specimen, LIM: Orrie, The Downs, Baragwanath Pass, forest edge, 24°08'S, 29°57'E, 900–1370 m, 14.iii.1998, M. Biondi & M. Zapparoli leg. (BAQ); 2 specimens, Mpumalanga: Mariepskop base Picnic Site at Blyde River, –24.5931S/30.8249E [24°35'35"S, 30°49'29"E], 780 m, night collecting, 26.xi.2012, R. Ruta leg. (UWCP); 1 specimen, Transvaal [MPU]: Blydepoort [24°34'51"S, 30°46'20"E], 20.xi.1981, Klapperich leg. (BAQ); 1 specimen, Transvaal [MPU]: Pretoriuskop, 25°10'S, 31°16'E, 500 m, 12.xi.1988, E. Colonnelli leg. (BAQ); 7 specimens, Transvaal [MPU]: Badplaas, 26°03'S, 30°33'E, 1250 m, 25.xi.1988, E. Colonnelli leg. (BAQ); 2 specimens, MPU: Mapoch’s Caves, c. 4 km ENE Roossenekal, 25°11'S, 29°58'E, 16.i.1989, collected from *Rhus* sp. (Anacardiaceae), E. Grobbelaar leg. (SANC); 10 specimens, Tvl. [MPU]: 20 km NE of Barberton, 25°41'S, 31°09'E, 21.iii.1993, collected from *Rhuspentheri* Zahlbr. (Anacardiaceae), E. Grobbelaar leg. (SANC); 5 specimens, Tvl. [MPU]: Sudwala Caves, [N]W of Nelspruit, 25°22'S, 30°42'E, 21.iii.1993, E. Grobbelaar leg. (SANC); 12 specimens, MPU: Paddadors Farm, Nelspruit, 22 km SE, 25°37'02"S, 31°07'56"E, 28.i.1984, E. de Wet, A. Nel & E. Grobbelaar leg. (SANC); 1 specimen, [MPU]: Marloth Park, 25°21'S, 31°47'E, 04.iv.1989, F.J. Joubert leg. (SANC); 1 specimen, Tvl. [MPU]: Swadini, Blydepoort Nat.[ure] Res.[erve], 24°32'S, 30°54'E, 26–29.i.1987, collected by beating, B.[=E.] Grobbelaar leg. (SANC); 1 specimen, Tvl. [MPU]: Barberton, 25°48'S, 31°03'E, 26–29.iii.1979, C. Moolman leg. (SANC); 1 specimen, ibid, iii.1979, C. Kok leg. (SANC); 1 specimen, MPU: Gustav Klingbiel Nature Reserve, 25°06'S, 30°00'E, 17.i.1989, collected by sweeping, E. Grobbelaar leg. (SANC); 5 specimens, TVL [MPU]: Blyderivierpoortdam Nat.[ure] Reserve, 24°32'S, 30°47'E, 25–26.x.1984, G.L. Prinsloo leg. (SANC); 1 specimen, Tvl [MPU]: between Baberton & Kaap Muiden, 24°29'S, 28°35'E, 25.ii.1991, V.M. Uys leg. (SANC).

#### Taxonomic remarks.

*Calothecanigromaculata* displays much variation in the number, shape, and color of the elytral patches (Fig. 2A–C), and in some biometric ratios (e.g., LE/LP) (Fig. 4). However, pronotal shape, sculpture, and color are consistent and useful for identification (Fig. 2A–D): lateral margins barely or not visible in dorsal view, more incurved in the anterior third; punctate lateral striae and basal furrows distinctly impressed and generally darker than the rest of the pronotal surface; pronotal margins mostly darkened. Median lobe of the aedeagus (Fig. 2F) in ventral view: lateral margins sinuate, but prominently rounded in apical 1/4, subtruncate apically; ventral surface with a pair of rounded lateral U-shaped depressions with a wrinkled surface in the apical half; surface clearly punctate in the apical 1/4; in lateral view, aedeagus curved in the basal 1/2 and distinctly sinuate in the apical 1/2; dorsal ligula short but clearly visible in lateral view, formed by a subtriangular, apically truncate median lobe, and two lateral lobes. The apical part of the median lobe shows considerable variability: in ventral view it is more or less sinuate laterally and more or less prominently rounded in apical 1/4, and more or less sinuate in lateral view. Spermatheca (Fig. 2E) globosely fusiform basally, sub-conical and generally dorsally orientated at the ductus attachment; distal part distinctly curved, generally about as long as the basal part, with a distinct appendix; ductus basally inserted, thickset, short, uncoiled, roughly U-shaped.

#### Biometrics.

Males (*n* = 10; mean ± standard deviation, range): LE = 5.04 ± 0.37 mm (4.25 ≤ LE ≤ 5.30 mm); WE = 3.73 ± 0.25 mm (3.18 ≤ WE ≤ 4.10 mm); LP = 1.46 ± 0.07 mm (1.35 ≤ LP ≤ 1.55 mm); WP = 2.88 ± 0.20 mm (2.45 ≤ WP ≤ 3.10 mm); LAN = 2.74 ± 0.15 mm (2.45 ≤ LAN ≤ 3.00 mm); LAED = 2.62 ± 0.10 mm (2.45 ≤ LAED ≤ 2.83 mm); LB = 6.36 ± 0.53 mm (5.15 ≤ LB ≤ 7.15 mm); LE/LP = 3.45 ± 0.21 (3.19 ≤ LE/LP ≤ 3.79); WE/WP = 1.30 ± 0.03 (1.24 ≤ WE/WP ≤ 1.36); WP/LP = 1.97 ± 0.08 (1.81 ≤ WP/LP ≤ 2.07); WE/LE = 0.74 ± 0.02 (0.71 ≤ WE/LE ≤ 0.78); LAN/LB = 0.43 ± 0.03 (0.39 ≤ LAN/LB ≤ 0.48); LE/LAED = 1.92 ± 0.09 (1.73 ≤ LE/LAED ≤ 2.00). Females (*n* = 10; mean ± standard deviation; range): LE = 5.41 ± 0.37 mm (4.75 ≤ LE ≤ 6.00 mm); WE = 3.96 ± 0.28 mm (3.40 ≤ WE ≤ 4.30 mm); LP = 1.42 ± 0.12 mm (1.23 ≤ LP ≤ 1.55 mm); WP = 3.01 ± 0.25 mm (2.70 ≤ WP ≤ 3.25 mm); LAN = 2.60 ± 0.21 mm (2.20 ≤ LAN ≤ 2.93 mm); LSP = 0.70 ± 0.06 mm (0.63 ≤ LSP ≤ 0.85 mm); LB = 6.48 ± 0.48 mm (5.60 ≤ LB ≤ 7.25 mm); LE/LP = 3.81 ± 0.17 (3.57 ≤ LE/LP ≤ 4.00); WE/WP = 1.32 ± 0.03 (1.26 ≤ WE/WP ≤ 1.37); WP/LP = 2.12 ± 0.06 (2.03 ≤ WP/LP ≤ 2.25); WE/LE = 0.73 ± 0.01 (0.72 ≤ WE/LE ≤ 0.77); LAN/LB = 0.40 ± 0.02 (0.38 ≤ LAN/LB ≤ 0.44); LE/LSP = 7.71 ± 0.33 (7.48 ≤ LE/LSP ≤ 8.23).

#### Distribution.

Mozambique and the Republic of South Africa (KZN, LIM, MPU). Records from Namibia (1 specimen, Fish River Canyon, Ai-Ais, 27°55'S, 17°29'E, 250 m, 13.ii.1994, F. Koch leg. (ZSM)), WCape Province (RSA) (3 specimens, Knysna, [34°02'S, 23°03'E], i.1979, C.D. Eardley leg. (SANC)), and Tanzania (Biondi et al. 2017) need additional confirmation (Fig. 5). Chorotype: probably Southern-Eastern Afrotropical (SEA).

#### Ecological notes.

Adults were collected from October to March, between 42–1650 m a.s.l., on *Searsia* sp. [= *Rhus* pars, cf. Moffett (2007)], *S.leptodictya* (along with larvae), S.cf.gueinzii, *S.pentheri* (Anacardiaceae), and on *Allophylusdecipiens* (Sapindaceae), in forest or habitat with very short grass.

### ﻿ Calotheca wanati sp. nov.

Taxon classificationAnimaliaPoalesPoaceae

30277C33-8507-52BF-98FA-91464680D5E9

http://zoobank.org/36AF9FD0-098F-4444-ABF5-AF7CEA799A5D

Figures 3A–D , 4 , 5

#### Type material.

***Holotype*** ♂: Republic Of South Africa: KZN: Ubombo Mountain Nat. Res., –27.6100S/32.0802E [27°36'36"S, 32°04'49"E], 110 m, beating, 30.xi.2012, M. Wanat leg. (SANC). ***Paratypes***: Republic Of South Africa: 3♂8♀, same data as for holotype (UWCP); 1♀, ibid, R. Ruta leg. (UWCP); 1♂1♀, KZN: Jozini, 10 km SW, W slope of Ubombo Mts, 27°28'S, 32°01'E, 500 m, 23.i.2006, adults beaten off *Allophylusnatalensis* (Sapindaceae), E. Grobbelaar leg. (SANC); 2♂2♀, KwaZulu-Natal: Mkhuze Game Res., –27.6392S/32.1583E [27°38'21"S, 32°09'29"E], 100 m, camping site, beating, 1.xii.2012, M. Wanat leg. (UWCP); 1♀, KwaZulu-Natal, Sodwana Bay, –27.5315S/32.6699E [27°31'53"S, 32°40'12"E], 5 m, swamp forest, site 1, 3.xii.2012, M. Wanat leg. (UWCP).

#### Diagnosis.

*Calothecawanati* sp. nov. is very similar to *C.nigromaculata*, both in external morphology and shape of the aedeagus and spermatheca. It is mainly distinguishable by: the almost invariable elytral color pattern, with quite small, irregular, light brown patches (*C.nigromaculata* is very variable in number, shape, and color of elytral patches, but the color pattern is generally different from *C.wanati* sp. nov.) (Figs 2A–C, 3A); the first pro- and metatarsomeres in male less enlarged, slightly larger than the distal part of the tibia (distinctly larger than the distal part of the tibia in *C.nigromaculata*) (Figs 2A–C, 3A); and the female has enlarged elytra (WE/LE = 0.76 ± 0.01 in *C.wanati* sp. nov.) (WE/LE = 0.73 ± 0.01 in *C.nigromaculata*) (Fig. 4B). The aedeagus is relatively short in *C.wanati* sp. nov. (LE/LAED = 2.16 ± 0.01) (Fig. 4C), in ventral view it has a sub-rhomboidal apical 1/4, and the prominent lateral expansions are angulate; in lateral view the median lobe is more distinctly curved apically (in *C.nigromaculata*, it is more elongate–LE/LAED = 1.92 ± 0.09, with indistict or very indistinct rounded lateral expansions in apical 1/4 in ventral view, and only slightly curved apically in lateral view) (Figs 2F, 3B).

#### Description of the holotype (♂).

Body elliptical in dorsal view (Fig. 3A), rather convex in lateral view; total length of the body (LB) = 6.40 mm; maximum pronotal width in the middle (WP = 2.98 mm); maximum width of elytra in the basal third (WE = 3.80 mm). Head pale brown, slightly darker along the frontal grooves; antennae pale brown, with antennomeres 6–11 slightly darkened (Fig. 3A); surface microreticulate and densely micropunctate, with evident setiferous punctures on most of the vertex and part of the frons; frontal grooves sinuate, very deeply impressed, extending from approximately the dorsal ocular margin to the inter-antennal space (Fig. 3C); eyes elliptical, clearly elongate; antennae shorter than half the body length (LAN = 2.75 mm; LAN/LB = 0.43); LA: 100:50:61:61:67:67:61:61:64:61:100. Pronotum (Fig. 3A, C) slightly convex, pale brown, punctate lateral stria and part of the margins darkened; distinctly transverse in dorsal view (LP = 1.40 mm; WP/LP = 2.13), sub-rectangular, with sides more distinctly incurved in the anterior third; surface microreticulate and micropunctate, with minute punctation; anterolateral surface with very shallow depressions; punctate lateral striae distinctly impressed, C-shaped, and slightly curved on the disc; some additional sparse punctures along the pronotal margins; basal furrows short and moderately incised; anterior, basal, and lateral margins evenly and finely bordered; lateral margins barely visible in dorsal view; anterior angles distinctly prominent, indistinctly swollen; posterior angles obtuse. Scutellum brown, sub-triangular. Elytra (Fig. 3A) elongate (LE = 4.90 mm; WE/LE = 0.78; LE/LP = 3.50), moderately rounded and indistinctly sinuate laterally, jointly rounded apically; slightly paler than pronotum, with irregular brown patches and brown punctation; lateral margin narrow, barely visible in dorsal view; elytral punctation arranged in single regular rows formed by distinctly impressed punctures; interstriae flat on the elytral disc, with finely microreticulate and sparsely micropunctate surface; humeral calli indistinctly raised. Macropterous. Basal pro- and mesotarsomeres clearly enlarged (Fig. 3A). Underside brown; apical abdominal ventrite without preapical sculptures or impressions. Median lobe of the aedeagus (LAED = 2.28 mm; LE/LAED = 2.15) (Fig. 3B) distinctly sinuate laterally in ventral view; in ventral view it has a sub-rhomboidal apical 1/4 truncate apically, and the prominent lateral projections are angulate; ventral surface with a pair of elongate lateral U-shaped depressions with a wrinkled surface in the apical half; surface distinctly punctate in the apical 1/4; aedeagus curved in the basal 1/2 and distinctly sinuate in the apical 1/2; dorsal ligula short but clearly visible in lateral view, formed by a subtriangular apically truncate median lobe, and two narrower lateral lobes.

#### Variability.

Males (*n* = 7; mean ± standard deviation, range): LE = 4.94 ± 0.12 mm (4.75 ≤ LE ≤ 5.10 mm); WE = 3.80 ± 0.09 mm (3.65 ≤ WE ≤ 3.90 mm); LP = 1.45 ± 0.05 mm (1.38 ≤ LP ≤ 1.50 mm); WP = 2.95 ± 0.06 mm (2.85 ≤ WP ≤ 3.03 mm); LAN = 2.66 ± 0.10 mm (2.50 ≤ LAN ≤ 2.75 mm); LAED = 2.28 ± 0.06 mm (2.20 ≤ LAED ≤ 2.35 mm); LB = 6.25 ± 0.22 mm (6.00 ≤ LB ≤ 6.60 mm); LE/LP = 3.40 ± 0.11 (3.27 ≤ LE/LP ≤ 3.53); WE/WP = 1.29 ± 0.01 (1.28 ≤ WE/WP ≤ 1.31); WP/LP = 2.03 ± 0.06 (1.97 ≤ WP/LP ≤ 2.13); WE/LE = 0.77 ± 0.01 (0.75 ≤ WE/LE ≤ 0.79); LAN/LB = 0.43 ± 0.02 (0.38 ≤ LAN/LB ≤ 0.45); LE/LAED = 2.16 ± 0.07 (2.02 ≤ LE/LAED ≤ 2.22). Females (*n* = 10; mean ± standard deviation; range): LE = 5.43 ± 0.25 mm (5.20 ≤ LE ≤ 5.70 mm); WE = 4.13 ± 0.19 mm (3.75 ≤ WE ≤ 4.30 mm); LP = 1.54 ± 0.08 mm (1.40 ≤ LP ≤ 1.55 mm); WP = 3.14 ± 0.13 mm (2.88 ≤ WP ≤ 3.28 mm); LAN = 2.61 ± 0.18 mm (2.40 ≤ LAN ≤ 3.05 mm); LSP = 0.66 ± 0.04 mm (0.60 ≤ LSP ≤ 0.75 mm); LB = 6.68 ± 0.34 mm (6.05 ≤ LB ≤ 7.10 mm); LE/LP = 3.53 ± 0.11 (3.35 ≤ LE/LP ≤ 3.70); WE/WP = 1.31 ± 0.02 (1.30 ≤ WE/WP ≤ 1.36); WP/LP = 2.04 ± 0.07 (1.88 ≤ WP/LP ≤ 2.13); WE/LE = 0.76 ± 0.01 (0.74 ≤ WE/LE ≤ 0.78); LAN/LB = 0.39 ± 0.02 (0.36 ≤ LAN/LB ≤ 0.45); LE/LSP = 8.21 ± 0.28 (7.93 ≤ LE/LSP ≤ 8.54). Paratypes very similar in shape, sculpture, and color to the holotype. Maximum pronotal width close to the pronotal base in some specimens. Female with basal pro- and mesotarsomeres less enlarged than in male. Spermatheca (Fig. 3D) globosely fusiform basally, subconical at the ductus attachment; distal part distinctly curved and slightly shorter than the basal part, with a distinct, irregularly enlarged appendix; ductus basally inserted, thickset, short, uncoiled, roughly U-shaped.

#### Etymology.

The specific epithet is a noun in the genitive case after Dr Marek Wanat (University of Wrocław, Poland), one of its collectors and esteemed expert of ColeopteraCurculionoidea.

#### Distribution.

Republic of South Africa (KZN) (Fig. 5). Chorotype: Southern-Eastern African (SEA).

#### Ecological notes.

Adults were collected in November, December, and January, between 5–500 m a.s.l., on *Allophylusnatalensis* (Sapindaceae) on one occasion and in swamp forest during a different collecting event.

## ﻿Discussion

*Calothecacarolineae* sp. nov., *C.nigromaculata* and *C.wanati* sp. nov. differ from the other known *Calotheca* species in that they share a combination of morphological characters, which are listed below. The basal part of the spermatheca is sub-fusiform; the area where the ductus is attached is roughly conical; the distal part is distinctly curved, elongate and about 2/3 or sub-equal to the basal part in length, with a distinct appendix; the ductus is basally inserted, quite thickset, short, uncoiled, and roughly U-shaped (Figs 1D, 2E, 3D). Ventrally the aedeagus has a pair of elongate to sub-rounded U-shaped depressions with a wrinkled surface in the distal 1/2, and distinct punctation towards the apex (Figs 1B, 2F, 3B). The pronotum shows distinct, but not expanded, lateral margins which are not thicker than the basal margin, barely or not visible in dorsal view, and more distinctly incurved in the anterior third; the punctate lateral striae, and sometimes the basal furrows, are darker in color than the rest of the pronotal surface (Figs 1C, 2D, 3C). The head has evident setiferous punctures on most of the surface of the vertex and at least on part of the frons (Figs 1C, 2D, 3C). Clear similarity in the shape of the median lobe of the aedeagus of *C.nigromaculata* and *C.wanati* sp. nov. reveals a closer affinity between these species than with *C.carolineae* sp. nov. In these two species, the aedeagus shows (Figs 2F, 3B): in ventral view the apical 1/4 sub-rhomboidal, truncate, or slightly rounded apically, with a strongly punctate surface; two lateral U-shaped depressions with a wrinkled surface in the distal half; in dorsal view a short ligula, with a slender median lobe and lateral lobes; in lateral view the apical part clearly sinuate. The main characters discussed above are reported below in the form of a key to species to facilitate the identification of the specimens.

Based on the available data, *C.nigromaculata* displays a wider distribution, that includes those of *C.carolineae* sp. nov. and *C.wanati* sp. nov. The species are even syntopic in some areas (*C.nigromaculata* with *C.wanati* sp. nov. in Ubombo Mountain Nature Reserve, and *C.wanati* sp. nov. with *C.carolineae* sp. nov. in Sodwana Bay), and they are associated with the same plant genus *Allophylus* (Sapindaceae), with *C.nigromaculata* also collected on *Searsia* (Anacardiaceae). Sapindaceae represents the first record of a host plant family other than Anacardiaceae. The two families belong to the same order Sapindales (The Angiosperm Phylogeny Group 2016), indicating a possible phylogenetically constrained host-use for *Calotheca*.

### ﻿Key to species of the *Calothecanigromaculata* group

The three species are distinguishable mainly by the characters of the median lobe of the aedeagus. Females can be identified by evaluating the combination of: color pattern, which is consistent within *Calothecacarolineae* sp. nov. and *C.wanati* sp. nov. but variable in *C.nigromaculata*; pronotal shape; some biometric features, such as LAN/LB and WE/LE.

**Table:** 

1	Apical part of the median lobe of the aedeagus widely rounded in ventral view, and not sinuate in lateral view (Fig. 1B). Pronotum straight laterally in basal 2/3 and abruptly incurved in the apical 1/3 (Fig. 1C). Dorsal integuments with clearly defined and generally larger elytral patches, reddish-brown on yellow surface (Fig. 1A). Antennae generally longer: LAN/LB > 0.45 in male and > 0.40 in female	***Calothecacarolineae* sp. nov.**
–	Apical part of the median lobe of the aedeagus sub-rhomboidal, and sub-truncate apically in ventral view; clearly sinuate towards apex in lateral view (Figs 2F, 3B). Pronotum slightly curved laterally in basal 2/3 and more distinctly incurved in the apical 1/3 (Figs 2D, 3C). Dorsal integument generally with smaller and more confused patches, from brown to black (Figs 2A–C, 3A). Antennae generally shorter: LAN/LB ≤ 0.45 in male and ≤ 0.40 in female	**2**
2	Median lobe of aedeagus shorter (LE/LAED > 2) (Fig. 4C), sub-rhomboidal apical 1/4 with prominent angulate lateral projections in ventral view; in lateral view, median lobe more distinctly curved apically (Fig. 3B). First pro- and metatarsomeres in male less enlarged, slightly larger than the distal part of the tibia. Female with more enlarged elytra (WE/LE generally > 0.74). Elytral patches quite small, irregular, light brown (Fig. 3A)	***C.wanati* sp. nov.**
–	Median lobe of aedeagus longer (LE/LAED ≤ 2) (Fig. 4C), sub-rhomboidal apical 1/4 with rounded, indistict or very indistinct lateral projections in ventral view; in lateral view, median lobe slightly curved apically (Fig. 2F). First pro- and metatarsomeres in male distinctly larger than the distal part of the tibia. Female with less enlarged elytra (WE/LE ≤ 0.74). Elytral patches variable in number, shape, and color, but generally larger or darker (Fig. 2A–C)	***C.nigromaculata* (Jacoby)**

## ﻿Conclusion

The genus *Calotheca* currently comprises 34 species, including the two new species here described. Diagnostic characters at species level, based on morphology, are mainly found on the median lobe of the aedeagus, the pronotum, and in the color of the dorsal integument. The identification of particular species groups relies mainly on the characteristics of the spermatheca and pronotum (D’Alessandro et al. 2020, 2021; Biondi unpublished data). Based on these characteristics *C.carolineae* sp. nov., *C.nigromaculata*, and *C.wanati* sp. nov. are here attributed to the *C.nigromaculata* species group. While the geographic distribution of the new species and the new distributional data of *C.nigromaculata* do not expand the geographic range of the genus, data on the association of the three species with the genus *Allophylus* (Sapindaceae) widen the range of its trophic spectrum, previously known as being limited to the family Anacardiaceae. However, due to the affinity between Anacardiaceae and Sapindaceae (The Angiosperm Phylogeny Group 2016), *Calotheca* species feeding on both the plant families cannot be considered as polyphagous.

## Supplementary Material

XML Treatment for Calotheca carolineae

XML Treatment for Calotheca nigromaculata

XML Treatment for Calotheca wanati
